# Supplementary material for: Transcription factor RonA-driven GlcNAc catabolism is essential for growth, cell wall integrity, and pathogenicity in Aspergillus fumigatus
Source: Microbiol Spectr. 2025 Oct 8;13(11):e00122-25. doi: 10.1128/spectrum.00122-25 (PMC12584764; doi:10.1128/spectrum.00122-25)
Supplement: Supplemental figures — Figures S1 to S5. [file spectrum.00122-25-s0001.docx]

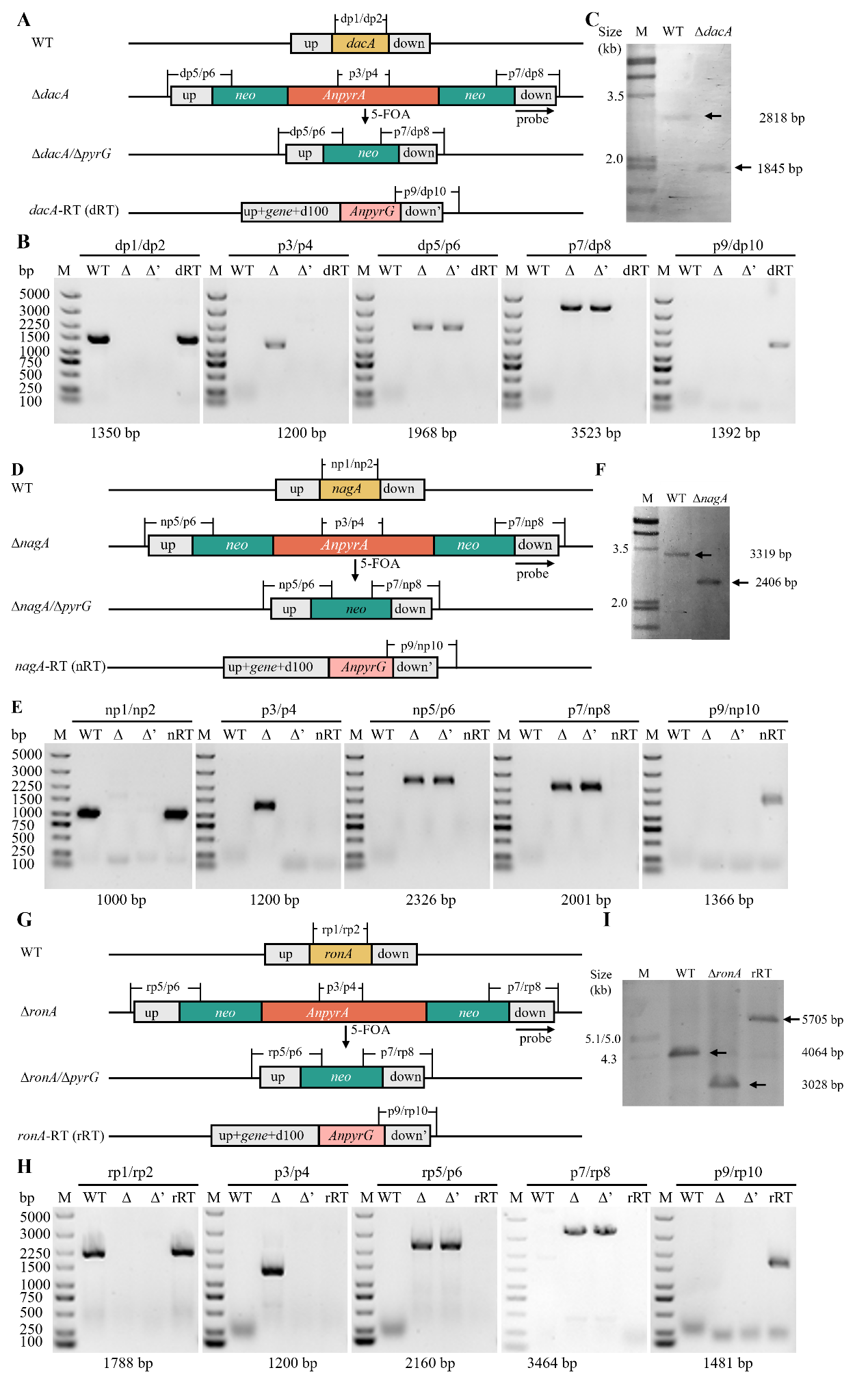


Supplementary Figure 1. **Validation of GlcNAc catabolism pathway mutants and revertant strains (RTs).** **(A, D, G)** Schematic representations of genetic manipulations and confirmation strategies for dacA (A), nagA (D), and ronA (G). **(B, E, H)** PCR-based genotyping of mutants and corresponding revertant strains: dacA, dacA/pyrG, and dRT (B); nagA, nagA/pyrG, and nRT (E); ronA, ronA/pyrG, and rRT (H). Genotyping was performed using five primer pairs per strain, as listed in Supplementary Table 1. **(C, F, I)** Southern blot analyses confirming genomic modifications: dacA mutant (C), nagA mutant (F), and ronA and rRT strains (I).


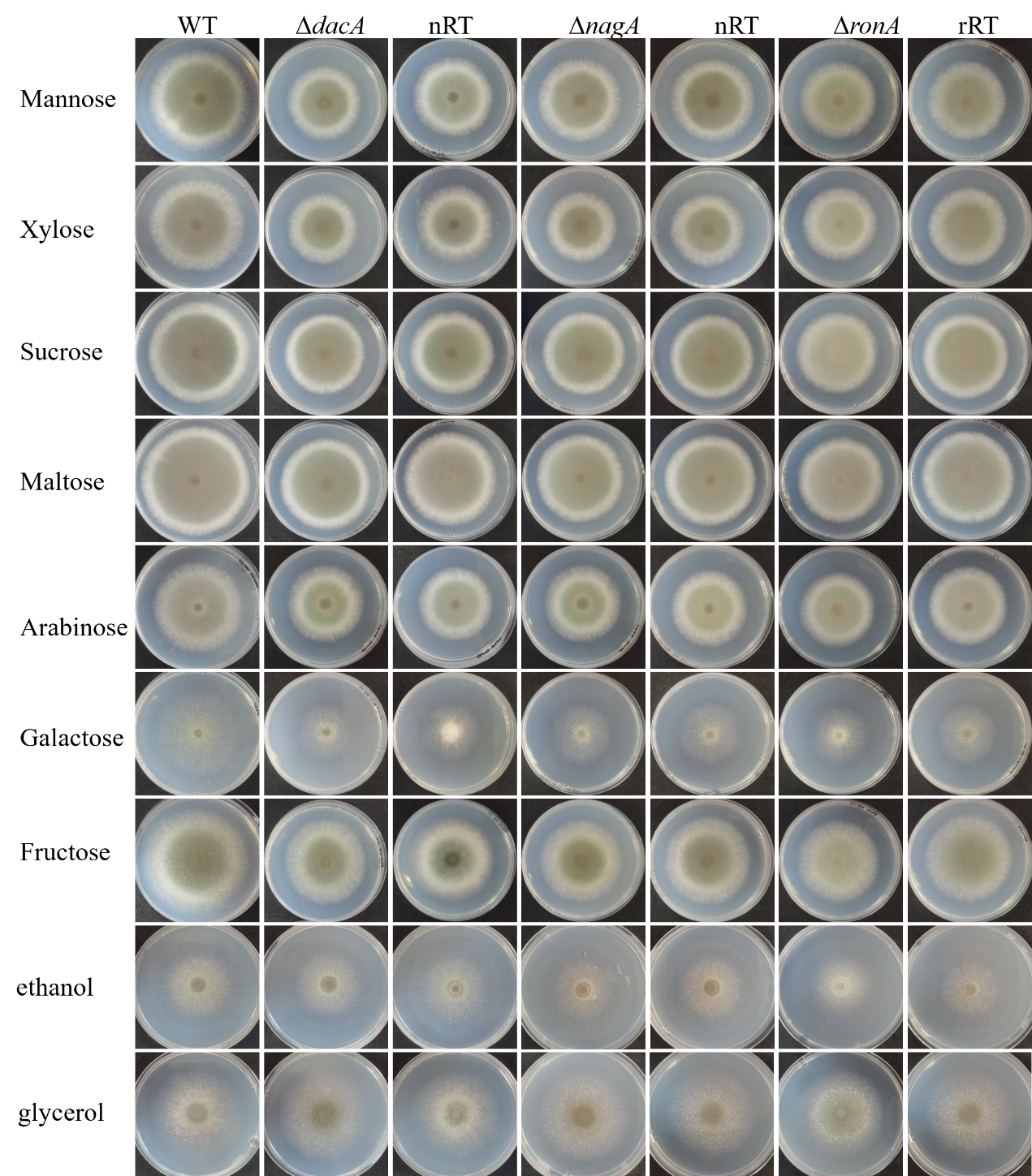


Supplementary Figure 2. Spot assays on different carbon sources. Spores (10^4^) of each strain were spotted on plates containing only mannose, xylose, sucrose, maltose, arabinose, galactose, fructose, ethanol or glycerol. Plates were photographed after 96 h incubation at 37 °C.


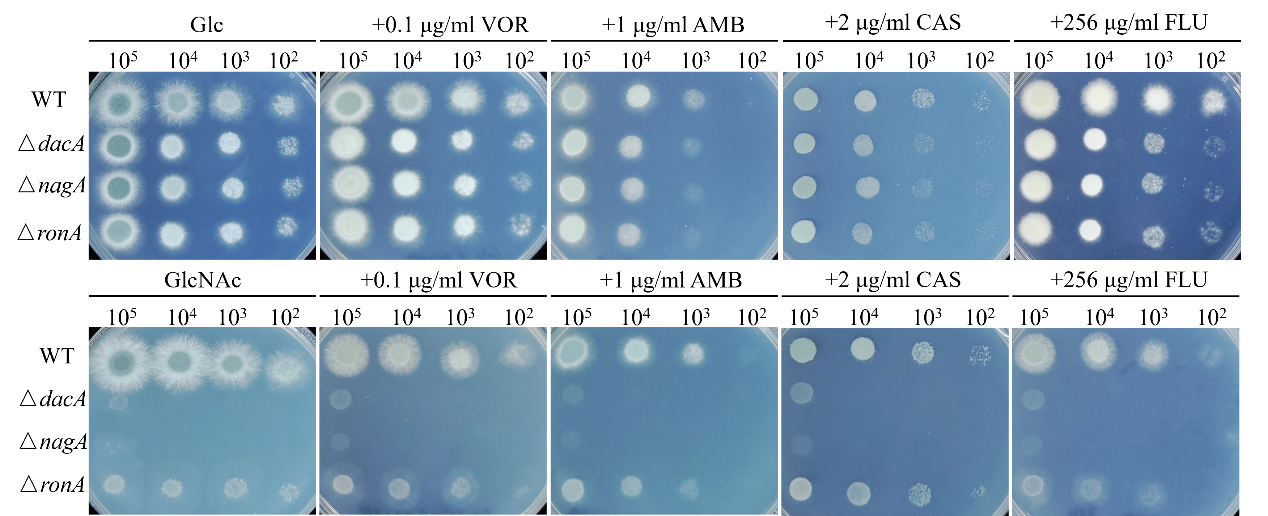


Supplementary Figure 3. Antifungal susceptibility of GlcNAc catabolism pathway mutants under Glc or GlcNAc conditions. Serial dilutions (10^5^-10^2^) of WT strain, Δ*dacA,* Δ*nagA* and Δ*ronA* mutants were spotted onto solid media plates containing either glucose or GlcNAc as the sole carbon source. Growth was assessed following 48 h incubation at 37°C. VOR: voriconazole, AmB: amphotericin B, CAS: caspofungin, FLU: fluconazole.


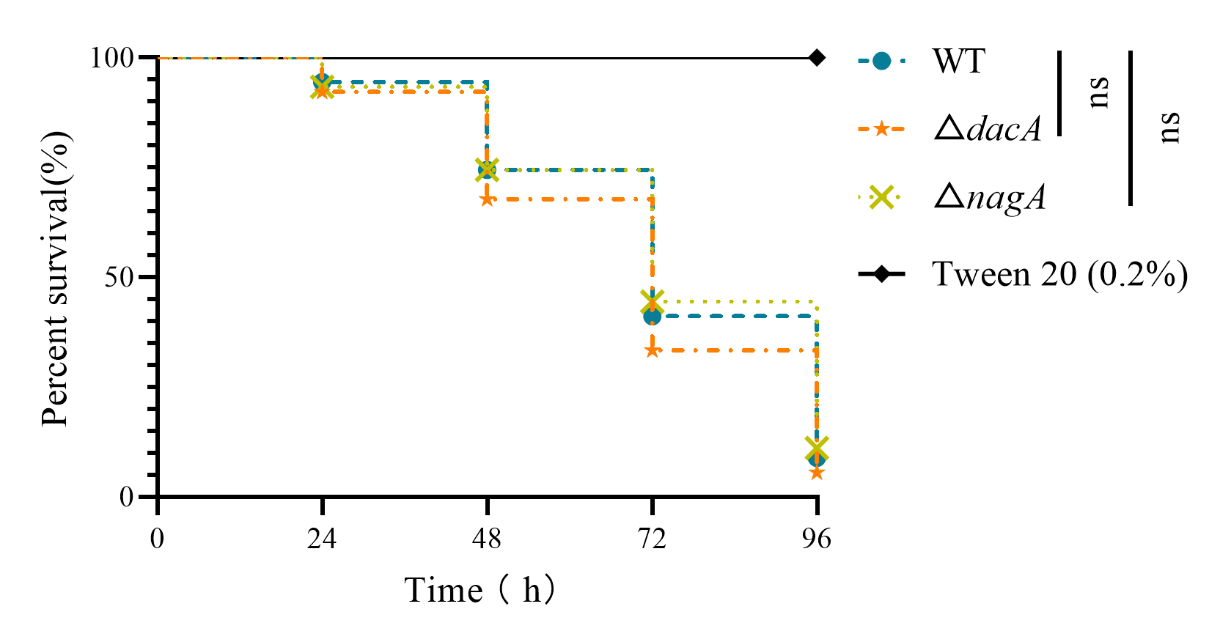


Supplementary Figure 4. Neither deletion of *dacA* nor *nagA* attenuates virulence. (A) Kaplan-Meier curves of the survival rates of *G. mellonolla* larvae at 24, 48, 72, and 96 h post-conidial injection. Three biological replicates were conducted for each strain.


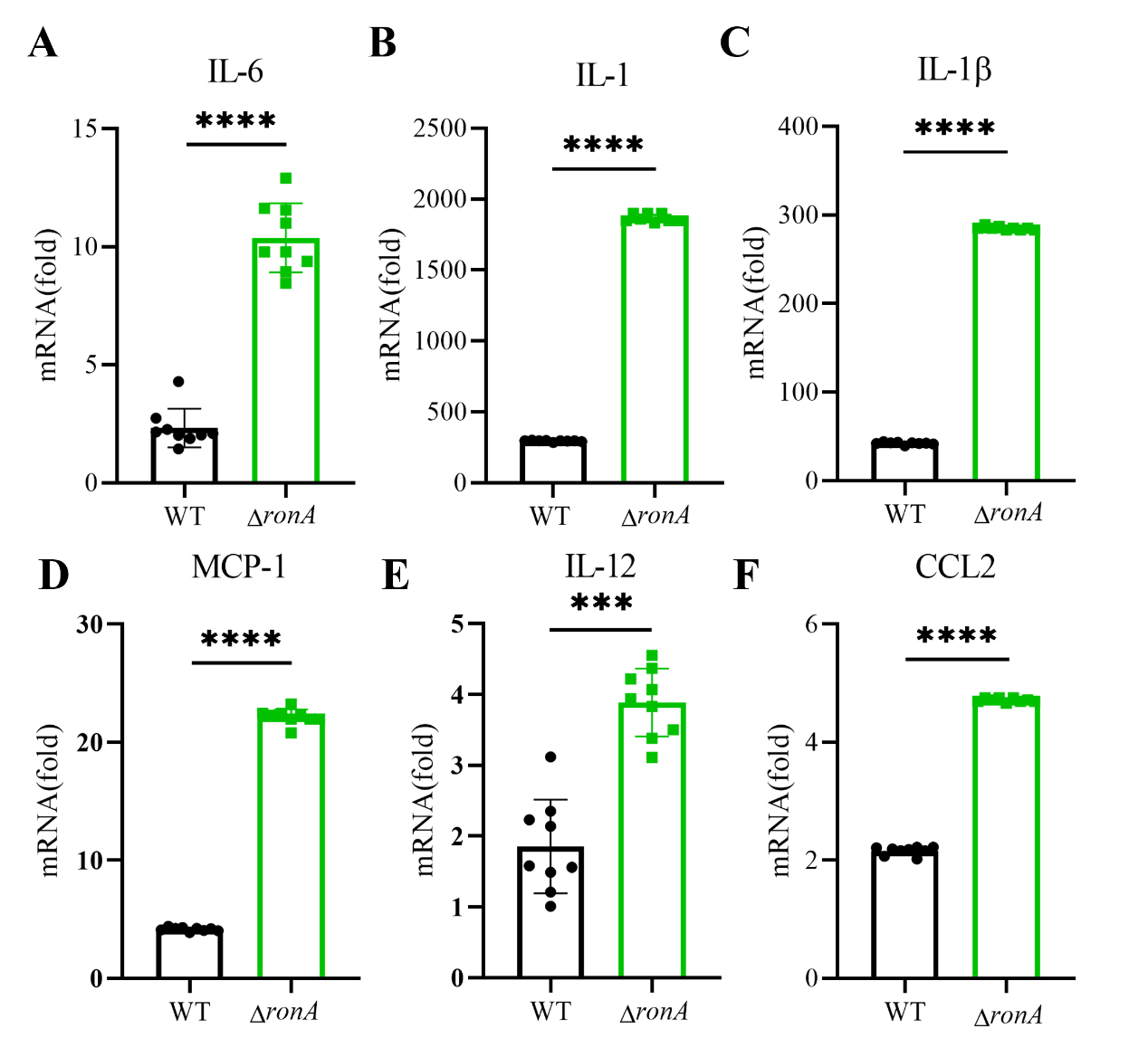


Supplementary Figure 5. Expression levels of **IL-6**, **IL-1α**, **IL-1β**, **MCP-1**, **IL-12**, **CXCL1**, and **CXCL2** in **RAW 264.7** macrophages infected with live WT or ronA mutant spores for 12 h. Gene expression was quantified by qRT-PCR using **9 biologically independent samples** (n = 9) and normalized to **β-actin** as the internal control.
